# Performance evaluation and reference interval establishment of Abbott Alinity thyroid-stimulating hormone receptor antibody (TRAb) assay for diagnosing Graves’ disease

**DOI:** 10.1371/journal.pone.0339494

**Published:** 2026-02-04

**Authors:** Hao Xue, Bowen Li, Xiaowei Wang, Junhan Zhao, Yaoming Yan, Jize Lao, Ling Ji, Yong Xia

**Affiliations:** 1 Department of Laboratory Medicine, Peking University Shenzhen Hospital, Shenzhen, China; 2 Core Diagnostics, Abbott Laboratories, Shanghai, China; Universidade dos Açores Departamento de Biologia: Universidade dos Acores Departamento de Biologia, PORTUGAL

## Abstract

**Objectives:**

This study aims to evaluate the analytical and clinical performance of the Abbott Alinity TRAb chemiluminescent microparticle immunoassay and establish the reference interval for healthy Chinese populations.

**Methods:**

The precision, analytical sensitivity and linearity of Abbott TRAb assay were verified in accordance with the Clinical and Laboratory Standards Institute guidelines. A total of 300 samples from patients with Grave’s disease (GD) and other thyroid diseases were collected for method comparison and clinical performance verification. The performance of Abbott and Snibe TRAb assays was compared to Roche TRAb assay by correlation and agreement analysis. The diagnostic performance of Abbott TRAb assay was analyzed via receiver operating characteristic (ROC) analysis. The reference interval of Abbott TRAb was established from a cohort of 366 healthy individuals.

**Results:**

The Abbott Alinity TRAb assay demonstrated excellent precision, with repeatability (CV%) ranging from 1.04% to 5.92%, and within-laboratory imprecision (CV%) ranging from 1.09% to 5.92%. Manufacturer-claimed limits of blank, detection, and quantification were successfully verified. Linearity was confirmed from 1.14 to 47.72 IU/L. Strong correlation was observed between Abbott and Roche assays (Spearman r = 0.972; slope = 1.060), while Snibe and Roche assays showed lower correlation (r = 0.784). Concordance analysis showed the total agreement with Roche results were 97.7% for Abbott and 96.7% for Snibe. Diagnostic accuracy of the Abbott TRAb assay for GD was high, yielding an area under curve (AUC) of 0.999, sensitivity of 98.4%, and specificity of 99.4% at an optimal cutoff of 2.89 IU/L. The upper reference limits at 95^th^ percentile and 97.5^th^ percentile for healthy Chinese population using Abbott TRAb assay were 1.56 IU/L and 1.95 IU/L, respectively.

**Conclusions:**

This study demonstrated the robustness of Abbott Alinity TRAb CMIA in clinical use with its verified analytical and clinical performance and established the reference interval for Chinese population.

## Introduction

Hyperthyroidism affects 0.5% to 2% of the global population [1]. In iodine-sufficient regions, the prevalence of overt hyperthyroidism ranges from 0.2% to 1.3%, with specific estimates of approximately 0.5% in the United States and a mean of 0.75% from a European meta-analysis [2]. Graves’ disease (GD) is the leading cause, accounting for 70% to 80% of hyperthyroidism cases [1,3,4]. GD exhibits a global prevalence of approximately 3% in women and 0.5% in men, with increased susceptibility among individuals aged 30–60 years [3,5]. In China, the prevalence of GD is 0.53%, which is comparable to that in developed countries [6]. GD is thought to result from a multifactorial interplay of genetic predisposition, environmental exposures, and immune dysregulation [7,8]. This pathological convergence induces the production of thyroid-stimulating hormone receptor (TSHR) autoantibodies, known as TRAb, which bind to TSHRs on the surface of thyroid follicular epithelial cells, mimicking the action of thyroid-stimulating hormone [7,8]. The continuous stimulation leads to hypertrophy and hyperplasia of the thyroid gland, resulting in elevated synthesis and secretion of thyroid hormones [7,8].

TRAbs are essential for the diagnosis and disease monitoring of GD [9]. Previous studies have shown that the positive rate of TRAb was higher in GD compared to other thyroid diseases, highlighting the value of TRAb in the diagnosis of GD [6]. Previous studies have consistently demonstrated that TRAb detection assays possess high diagnostic accuracy, with sensitivity exceeding 95% and specificity surpassing 97% [10–13]. Besides diagnosis, TRAb can also help to guide disease management. TRAb titers generally decrease after treatment [14], and a prospective study indicated that higher TRAb titers following antithyroid drug (ATD) therapy were associated with a 2.7-fold increased risk of GD recurrence [15]. Furthermore, a study conducted in a Chinese hospital found that patients with GD who had high TRAb levels at diagnosis were more likely to experience poor outcomes following ATD treatment [16].

TRAbs are classified as stimulating, blocking, or neutral, with stimulating antibodies being the most prevalent. TRAb detection methods include bioassays and competitive immunoassays [17]. Bioassays can evaluate functional activity of TRAb by measuring cyclic adenosine 3′,5′-monophosphate (cAMP) production in vitro, enabling the distinguishment between different TRAb subtypes [18,19]. Competitive immunoassays measure TRAb in serum samples by inhibiting the binding of TSHRs with known TSHR ligands but can’t distinguish between stimulating, blocking, or neutral TRAb[17]. Due to their automated design, competitive immunoassays are easy to perform and are faster compared to bioassays, which has led to their widespread use in clinical practice [17,20]. In 2003, a human monoclonal antibody to the TSHR (M22), was generated from the lymphocytes of a patient with GD [21]. This antibody, exhibiting strong thyroid-stimulating activity, facilitated the advancement of novel third-generation TRAb immunoassays [22,23].

Chemiluminescent immunoassay (CLIA) technology, a type of automated competitive immunoassay, offers rapid and accurate diagnosis of autoimmune diseases due to its wide dynamic range, high signal intensity, and specificity [24]. The Abbott TRAb chemiluminescent microparticle immunoassay (CMIA) uses CLIA technology and can be performed on the Abbott Alinity i platform [20]. Currently, Roche TRAb assay is widely used in China. It is a third-generation competitive electrochemiluminescence immunoassay (ECLIA) using the human monoclonal antibody M22, and is widely employed on Elecsys and Cobas e platforms for diagnosing GD due to its high sensitivity and specificity [10,25]. A previous study in Singapore showed Abbott Alinity I TRAb assay has close agreement with Roche Cobas e801 TRAb assay [20]. Snibe TRAb assay is another immunoassay to detect TRAb that has been used in China. It utilizes a sandwich CLIA on MAGLUMI analyzers, incorporating N-(4-Amino-Butyl)-N-Ethyl-Isoluminol (ABEI)-labeled reagents and TSHR-coated nanomagnetic microbeads to enhance analytical sensitivity and reduce performing time [26].

Although several studies have evaluated the analytical and clinical performance of several automated TRAb immunoassays among either Chinese or other population [27–30], the performance of Abbott TRAb CMIA assay has not been verified in China yet. Besides, a reliable population-based reference interval is crucial for making proper clinical decisions [31,32]. Previous studies have demonstrated that reference intervals can be influenced by factors such as gender, age, race, region, and detection methods [33,34]. Reference intervals for TRAb assays have been previously established in the Thai population, with 97.5^th^ percentiles ranging from 1.24 IU/L (Roche Cobas e411) to 1.67 IU/L (Roche Cobas e601) [35,36]. However, the reference interval for Abbott TRAb assay in Chinese population has yet to be established.

Therefore, this study aims to verify the analytical and clinical performance of the Abbott Alinity TRAb assay and compare it with the currently used TRAb assays in China, as well as to establish the reference interval for the healthy Chinese population.

## Materials and methods

### Analytical performance verification

Based on the guidance from Clinical and Laboratory Standards Institute (CLSI) document EP15-A3 [37], precision was assessed using Abbott Alinity TRAb controls at 3 different levels (3.0, 10.0 and 30.0 IU/L). These control levels represented concentrations near the manufacture-claimed cutoff value for diagnosing GD, moderate positive, and high positive values. Samples were tested according to a 5 × 1 × 5 scheme, meaning each sample underwent 25 tests over five days, with five replicates per day. Data was analyzed using EP Evaluator software (Data Innovation, USA) to determine the within-run and within-laboratory imprecision. Precision verification was considered successful when the within-run or within-laboratory standard deviation (SD) reported by the EP Evaluator was less than or equal to the verification value.

The Limit of Blank (LoB) and Limit of Detection (LoD) were verified following CLSI document EP17-A2 [38], while the Limit of Quantitation (LoQ) was verified according to CLSI document EP15-A3 guidelines [37].

For LoB and LoD verification, two blank samples and two samples with concentrations at the manufacturer claimed LoD (0.70 IU/L) were tested at least 20 times over five days. For LoB verification, success was defined by the criterion p ≥ P (p = $\frac{\text{Number}\;\text{of}\;\text{test}\;\text{results}\;\leq \;\text{LoB}}{\text{Total}\;\text{number}\;\text{of}\;\text{repeated}\;\text{tests}\;(\text{m})}$ × 100%) and P refers to the critical value observation ratio from supplementary material (S1 Table) corresponding to the total number of test results (N), with the closest N value used if m ≠ N). LoD verification followed the same principle, where success was defined by p ≥ P (p = $\frac{\text{Number}\;\text{of}\;\text{test}\;\text{results}\;\geq \;\text{LoB}}{\text{Total}\;\text{number}\;\text{of}\;\text{repeated}\;\text{tests}\;(\text{m})}$ × 100%) and P is determined as for LoB verification).

For LoQ verification, five samples at the claimed LoQ (1.26 IU/L) were tested at least 25 times over five days, with five replicates per day. The mean, standard deviation (SD), and coefficient of variation (CV%) for the 25 tests were calculated. If the calculated CV% was less than or equal to the precision requirement of 20% for the claimed LoQ, then the LoQ was considered verified. If the calculated CV% exceeded this precision requirement, the verification value of the precision-required SD was calculated using the formula: SD = manufacturer’s claimed LoQ × claimed CV%. The calculated SD was then compared with this verification value. When the calculated SD was less than or equal to the verification value, the claimed LoQ was verified; otherwise, it was not.

The linearity of TRAb was confirmed according to CLSI document EP06-A [39]. Dilutions at varying concentrations, prepared from two sample pools, were mixed in specific ratios and measured in triplicate. The preparation process involved creating a low-value sample plate with concentrations ranging from the lower limit of the manufacturer’s claimed measurement range to the lower limit plus 10%. Similarly, a high-value sample plate was prepared with concentrations spanning from 10% below the upper limit to the upper limit of the measurement range. Various samples with differing dilution levels were then prepared. The low-value sample plate was designated as Level 1, and the high-value sample plate as Level 5. For Level 2, 3 parts of Level 1 were mixed with 1 part of Level 5. Level 3 was prepared by mixing 2 parts of Level 1 with 2 parts of Level 5. Lastly, Level 4 was created by combining 1 part of Level 1 with 3 parts of Level 5. The mean test results for each sample level were calculated and plotted on the Y-axis against the theoretical concentrations on the X-axis, with each sample level marked. Polynomial regression analysis was used to assess linearity. When the nonlinear coefficients β0, β2, and β3 were not significantly different, the manufacturer claimed linearity was verified.

### Subjects and sample collection

Remnant serum samples from 150 patients diagnosed with GD and 150 patients with other thyroid diseases were collected for method comparison and clinical performance verification. For clinical performance verification, only samples from newly diagnosed patients who were treatment-naive were included to avoid the influence of medication on TRAb titers. The inclusion criteria for GD patients were: a) Age 18–75 years old; b) Diagnosis based on the 2022 edition of the “Guidelines for Diagnosis and Management of Hyperthyroidism and Other Causes of Thyrotoxicosis” in China; c) Patients with an initial diagnosis who have not received any treatment for GD. For other thyroid diseases (non-hyperthyroidism), the inclusion criteria were: a) Age 18–75 years old; b) Other clinically diagnosed thyroid diseases such as hypothyroidism, benign thyroid nodules, thyroid cancer, etc.

Additionally, serum samples from 366 healthy individuals were used to establish the reference interval. For healthy volunteers, the inclusion criteria were: a) Age 18–75 years old; b) Alanine aminotransferase (ALT), aspartate aminotransferase (AST), γ-glutamyltransferase (GGT), and total bilirubin levels below the upper limit of normal (ULN); c) Creatinine and blood urea nitrogen (BUN) levels below the ULN, with an estimated glomerular filtration rate (eGFR) >90 ml/min/1.73m^2^; d) No abnormalities in thyroid-stimulating hormone (TSH), free triiodothyronine (FT3), free thyroxine (FT4), total triiodothyronine (TT3), total thyroxine (TT4), antithyroglobulin antibody (TgAb), anti-thyroid peroxidase antibody (TPO-Ab), and fasting blood glucose; e) No history of thyroid cancer, thyroid nodules, autoimmune diseases, or other endocrine and metabolic diseases. Subjects were excluded if they had incomplete information, were pregnant or breastfeeding, had sample hemolysis, or had other conditions deemed unsuitable by the investigators.

Each subject’s remnant serum sample should be no less than 1 ml, with 500 μl/tube, divided into two tubes for freezing. The sample storage conditions were: a) After blood collection and before serum separation: room temperature <24 hours, 2–8 °C < 3 days; after serum separation: 2–8 °C < 3 days, −80 °C for long-term storage. This study was approved by the Research Ethics Committee of Peking University Shenzhen Hospital (Approval No. BDSYLS (Research) [2023] No. 168), which waived the requirement for informed consent. All patient data were anonymized to ensure confidentiality. Data access was limited to the period between March 8 and September 6, 2024, and was used solely for research purposes. Test results were not intended for clinical diagnosis, and no individual reports were issued. The study posed no risk to participants’ rights or health. Personal identifiers were removed through de-identification procedures. Medical records, source documents, and research data remained the property of the institution.

### TRAb measurement methods

The Alinity i system (Abbott Laboratories, USA) utilizes an automated, delayed one-step CMIA technology for quantitative measurement of TRAb in human serum [40]. A serum sample (50 μL) is combined with paramagnetic microparticles coated with mouse monoclonal IgG and an assay diluent containing recombinant human TSH receptor (rTSHR). This mixture is incubated for approximately 20 minutes, allowing TRAb in the patient sample to compete with the labeled M22 antibody for binding sites on the immobilized TSH receptor. TSH receptor M22 antibody acridinium-labeled conjugate is added to create a reaction mixture, which is then incubated for a further 5 minutes. Following a wash cycle, Pre-Trigger (hydrogen peroxide) and Trigger Solutions (sodium hydroxide) are added. The resulting chemiluminescent reaction is measured as a relative light unit (RLU). There is an indirect relationship between the amount of TRAb in the sample and the RLU detected by the system optics.

The Elecsys Anti-TSHR assay (Roche, Switzerland), conducted on Roche cobas e801 immunoassay analyzer, is a 27-minute automated ECLIA designed for quantitative determination of TRAb in human serum [41]. The procedure includes three incubation steps: 1) A 30 µL serum sample is combined with pretreatment buffers (ATSHR PT1 and PT2) to form an immunocomplex involving solubilized porcine TSH receptor (pTSHR) and a biotinylated mouse monoclonal antibody, allowing TRAb interaction; 2) An additional buffer enhances TRAb binding; 3) Streptavidin-coated microparticles and a ruthenium-labeled M22 monoclonal autoantibody are added to detect TRAb through its competitive inhibition of M22 binding. The immunocomplex is captured via biotin-streptavidin interaction and aspirated into a measuring cell, where microparticles are magnetically retained and unbound substances removed. Chemiluminescent emission is then induced and measured, with results calculated using a calibration curve generated through a 2-point system and a master curve provided.

The MAGLUMI TRAb (CLIA) test (Shenzhen New Industry, China), performed on a Fully-auto CLIA analyzer MAGLUMI X8, employs a sandwich immunoluminometric assay for the quantitative determination of TRAb in human serum [42]. The procedure begins with a 20 μL sample (patient sample, calibrator, or control) undergoing an auto-dilution step (1:11 dilution) with 200 μL of diluent. Subsequently, 20 μL of this auto-diluted sample, calibrator, or control is combined with 100 μL of Buffer and 20 μL of nano magnetic microbeads (coated with TSHR antigen, containing BSA and 0.2% NaN3). This mixture is incubated at 37°C for 10 minutes, followed by a cycle washing step using 400 μL of wash solution. Next, 100 μL of ABEI Label (SPA antigen labeled ABEI, containing BSA and 0.2% NaN3) is added, and the reaction mixture is incubated for an additional 10 minutes. A second washing cycle is then performed with 400 μL of wash solution. Finally, starter reagents are added to initiate a flash chemiluminescent reaction, and the resulting light signal, measured as RLU by a photomultiplier within 3 seconds, is directly proportional to the concentration of TRAb in the samples.

### Method comparison

The method comparison was conducted according to CLSI document EP09-A3 [43]. The 300 serum samples from patients with GD and other thyroid diseases were tested by Abbott Alinity i TRAb assay, Roche Elecsys anti-TSHR assay and Snibe MAGLUMI TRAb (CLIA) assay. Results obtained from Abbott TRAb assay and Snibe TRAb assay were compared with Roche TRAb assay respectively using Passing-Bablok regression analysis to assess linearity and proportional bias between methods. Bland-Altman plots were additionally constructed to evaluate the degree of agreement and systematic differences across assay pairs. Agreement metrics—including positive agreement, negative agreement, and total agreement—were calculated based on classification concordance relative to manufacturer-determined diagnostic cutoffs for TRAb.

### Clinical performance verification

The testing results for all 300 serum samples from patients with GD or other thyroid diseases using the Abbott Alinity i TRAb assays were utilized to generate a receiver operating characteristic (ROC) curve. The ROC curve was constructed using the resultant TRAb levels and their corresponding clinical diagnoses to assess the assay’s ability to distinguish GD cases from non-hyperthyroidism controls. The area under the ROC curve (AUC) was calculated to determine overall diagnostic accuracy. Optimal threshold values for TRAb were established using Youden’s index to simultaneously maximize sensitivity and specificity.

### Reference interval establishment

The reference interval were established using serum samples from healthy volunteers on the Abbott Alinity system, following CLSI document EP28-A3 guidelines [44]. TRAb measurement was performed on the Abbott Alinity i system following the manufacturer’s standard analytical protocols. Outliers were identified and removed using the Dixon method (D/R > 1/3). The intervals were set as unilateral upper reference limits. For normally distributed results, the upper reference limit was calculated using the formula μ + 1.64s (where μ is the mean and s is the standard deviation). For skewed distributions, the upper reference limit was determined using the 95^th^ or 97.5^th^ percentile with 95% confidence intervals.

### Statistical analysis

Data were presented as means where applicable. Pairwise agreement between test results from Abbott and Roche TRAb assay, as well as Snibe and Roche TRAb assay was assessed using Passing-Bablok analysis, respectively. Bias was evaluated with the Bland-Altman method. MedCalc Statistical Software version 23.0.2 (MedCalc Software bv, Ostend, Belgium) was used for the analysis. Polynomial regression was conducted for linearity analysis using Analysis ToolPak for Microsoft Excel. SPSS statistical software was used for ROC curve analysis and to assess the normality of experimental results in the establishment of the reference interval.

## Results

### Analytical performance

The precision results for the TRAb assay are detailed in Table 1. The repeatability (CV%) for samples at low, medium and high control levels were 0.139, 0.178, and 0.341, respectively, while the within-laboratory imprecision (CV%) were 0.151, 0.218, and 0.373, respectively.

**Table 1:** Precision results for TRAb assay expressed as imprecision (SD), obtained using three levels of Abbott controls.

Measurand	Level (IU/L)	With-in run imprecision			Within-laboratory imprecision			Interpretation
		SD	Verification Value	Manufacturer claimed CV%	SD	Verification Value	Manufacturer claimed CV%	
TRAb	3.0	0.139	0.182	4.80%	0.151	0.192	5.20%	Success
	10.0	0.178	0.233	1.80%	0.218	0.280	2.20%	Success
	30.0	0.341	0.446	1.10%	0.373	0.479	1.20%	Success

Abbreviations CV: imprecision; SD: standard deviation; TRAb: thyroid-stimulating hormone receptor antibody.

The manufacturer claimed the limits of blank (LoB), detection (LoD), and quantitation (LoQ) was 0.48 IU/L, 0.70 IU/L, and 1.26 IU/L, respectively. The percentages of test results at or below the claimed LoB and LoD were 100% and 98%, respectively. These values exceed the critical value observation ratio of 85%. The coefficient of variation (CV%) for LoQ verification was 14.3%, which did not exceed the acceptable imprecision limit of 20%.

**Fig 1:**
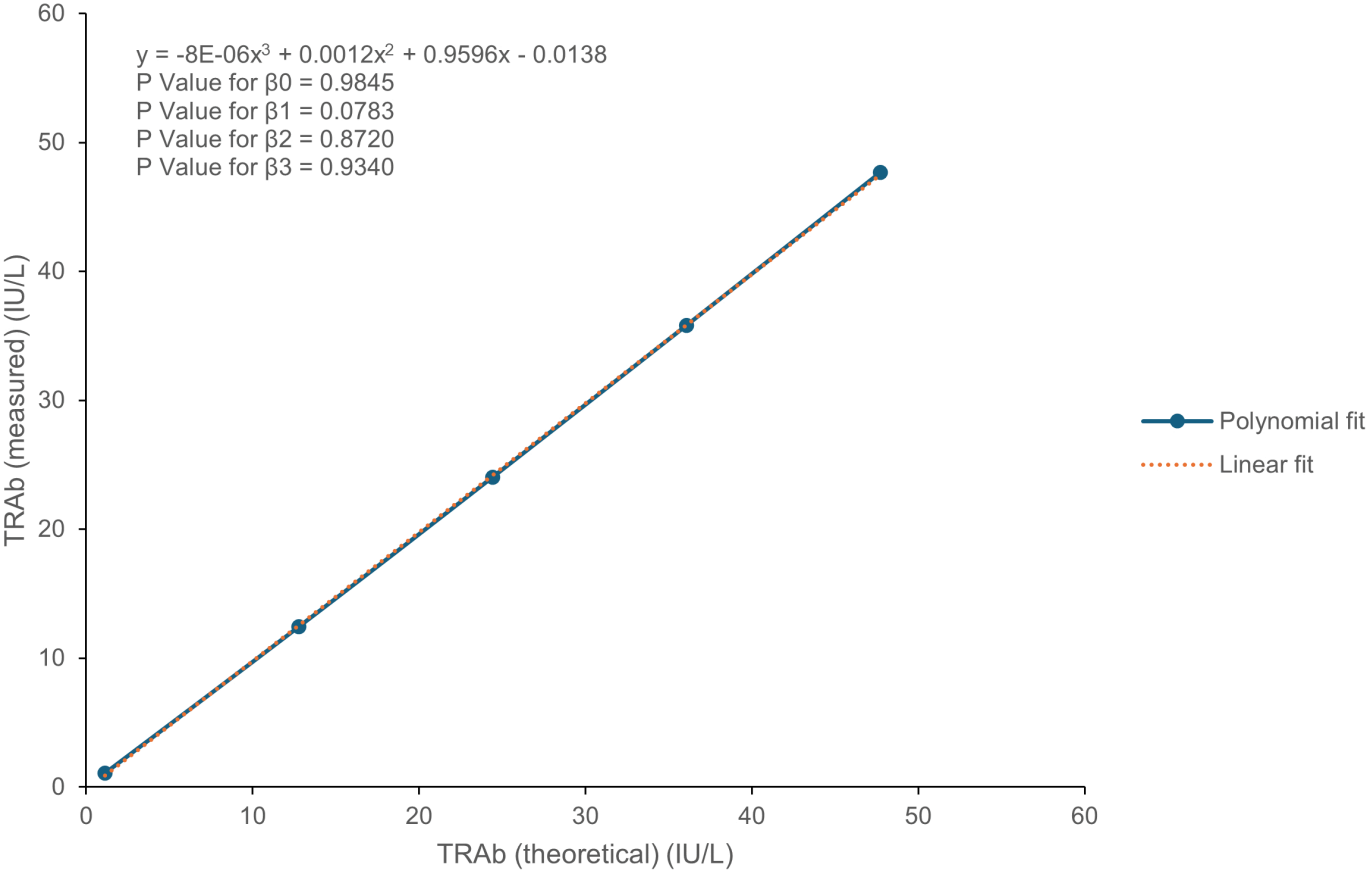
TRAb linearity results plotted against the theoretical TRAb levels using a polynomial cubic model.

Fig 1 illustrates the linearity of the TRAb assay. The assay passed the linearity verification from 1.14 to 47.72 IU/L, as the differences in the nonlinear coefficients β0, β2, and β3 were not statistically significant, with p-values of 0.9845, 0.8720, and 0.9340, respectively.

### Method comparison

**Fig 2:**
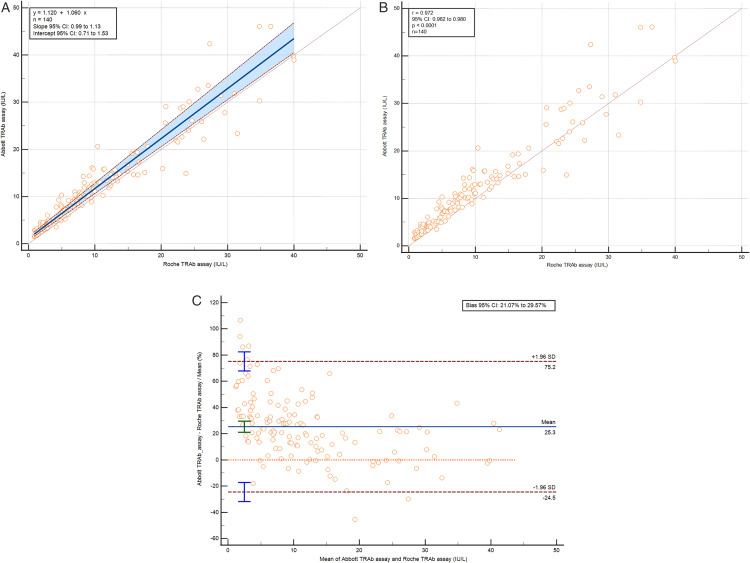
Method comparison results for TRAb (IU/L), limited to samples within the measuring range. **(A)** Passing-Bablok regression, **(B)** Spearman rank correlation test and **(C)** Bland-Altman analysis of Abbott vs. Roche.

A total of 300 serum samples from patients with GD (n = 150) and patients with other thyroid diseases (n = 150) were analyzed using Abbott, Snibe and Roche TRAb assays. The testing results range from 0.08 to >50.0 IU/L on Abbott platform, < 0.8 to >40.0 IU/L on Roche platform, and 0.175 to >30 IU/L on Snibe platform. For the Abbott vs. Roche TRAb assays, 140 results within both assays’ measuring ranges were evaluated using Passing-Bablok and Bland-Altman analyses. The y-intercept was 1.120, slope 1.060, with 95% CIs of 0.71 to 1.53 and 0.99 to 1.13. The Cusum test showed no significant deviation from linearity (p = 0.34), and the Spearman rank correlation coefficient was 0.972 (95% CI 0.962 to 0.980) with p < 0.0001 (Fig 2A and 2B). The Abbott TRAb assay had a positive bias of 1.83 IU/L (25.3%) compared to Roche (Fig 2C).

**Fig 3:**
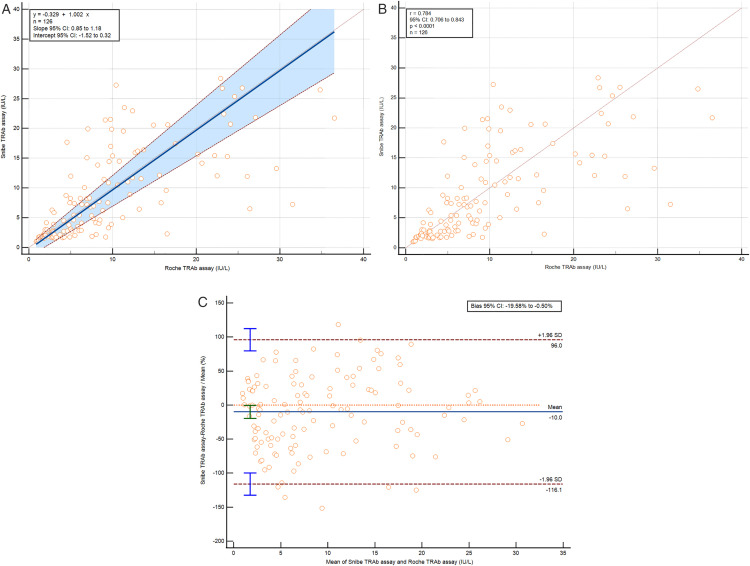
Method comparison results for TRAb (IU/L), limited to samples within the measuring range. **(A)** Passing-Bablok regression, **(B)** Spearman rank correlation test and **(C)** Bland-Altman analysis of Snibe vs. Roche.

For Snibe vs. Roche TRAb assays, 126 results were analyzed. The y-intercept was −0.329, slope 1.002, with 95% CIs of −1.52 to 0.32 and 0.85 to 1.18. The Cusum test showed no significant deviation from linearity (p = 0.19), and the Spearman rank correlation coefficient was 0.784 (95% CI 0.706 to 0.843) with p < 0.0001 (Fig 3A and 3B). The Snibe TRAb assay had a negative bias of −0.58 IU/L (−10.0%) compared to Roche (Fig 3C). The supplementary materials (S1 Fig and S2 Fig) illustrate the results of the subgroup pair-wise method comparisons for the samples from GD and non-GD patients.

Concordance analysis was performed on 300 serum samples using manufacturer-determined diagnostic cutoffs for Graves’ disease: 3.10 IU/L for the Abbott assay, 1.75 IU/L for the Roche assay, and 1.50 IU/L for the Snibe assay. For the Abbott vs. Roche TRAb assays, the positive agreement for 300 samples was 97.0% (131/135), the negative agreement was 98.2% (162/165), and the total agreement was 97.7% (293/300). Among the seven inconsistent results, four samples were negative for Abbott but positive for Roche and Snibe, with Abbott values being close to its cutoff values (2.54–3.09 IU/L). Three samples were positive for Abbott but negative for Roche, two of them were also positive for Snibe. Similarly, for the Snibe vs. Roche assays, the positive agreement was 100% (135/135), the negative agreement was 93.9% (155/165), and the total agreement was 96.7% (290/300). Of the ten inconsistent results, all were positive for Snibe (1.523–1.895 IU/L) but negative for Roche, and two were positive for Abbott. The details of the inconsistent results are provided in the supplementary materials (S2 Table and S3 Table).

### Clinical performance

**Fig 4:**
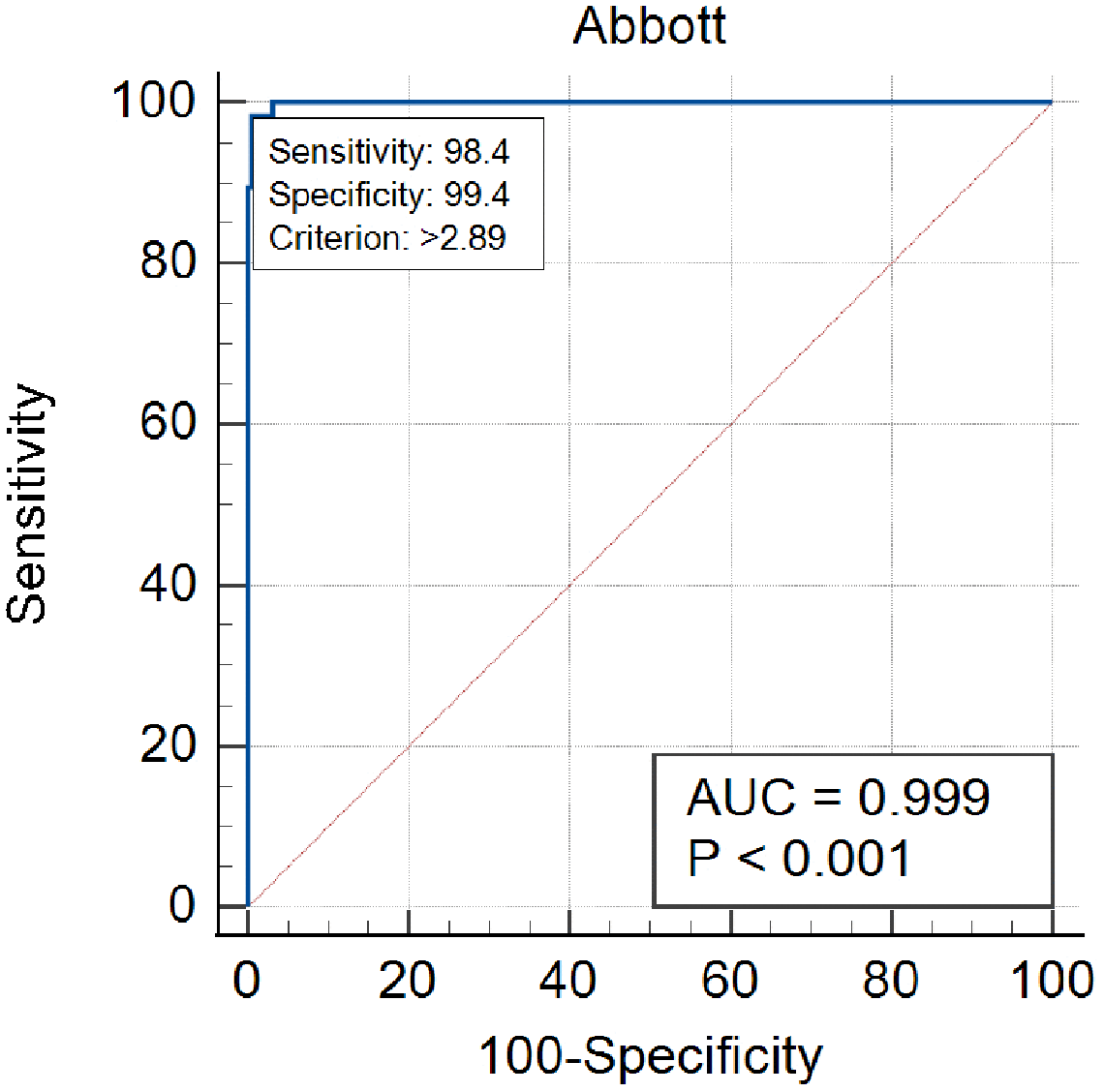
ROC curve analysis for the Abbott TRAb assay in diagnosing Graves’ disease.

Two hundred and eighty-seven samples out of 300 clinical samples were analyzed for clinical performance verification (124 from GD patients and 163 from patients with other thyroid diseases). The Abbott TRAb assay correctly identified 120 of the GD samples as positive, and 162 of the non-hyperthyroidism samples as negative (Table 2). The optimal cutoff value for Abbott TRAb assay was calculated to be 2.89 IU/L. With this optical cutoff value, the assay could achieve an AUC of 0.999 (95% CI 0.985–1.000, p < 0.001), a Youden index of 0.98, a sensitivity of 98.4%, and a specificity of 99.4% (Fig 4). The clinical performance was similar to manufacture’s claims (sensitivity of 96.8% and specificity of 99.4% with cutoff value set at 3.10 IU/L).

**Table 2:** Distribution of testing results for the Abbott Alinity TRAb assay in diagnosing Graves’ disease.

Abbott	Graves’ disease	Non-GD	Total
**TRAb (+)**	120	1	121
**TRAb (-)**	4	162	166
**Total**	124	163	287

### Reference interval

Table 3 presents the reference interval for the Abbott TRAb assay. Among the 366 volunteers, the upper limits were 1.56 IU/L (95% CI 1.45 to 1.78) at the 95^th^ percentile and 1.95 IU/L (95% CI 1.58 to 2.14) at the 97.5^th^ percentile. Among the 164 male volunteers, the upper limits were 1.51 IU/L (95% CI 1.40 to 2.00) at the 95^th^ percentile and 2.01 IU/L (95% CI 1.47 to 2.14) at the 97.5^th^ percentile. For the 201 female volunteers, the upper limits were 1.63 IU/L (95% CI 1.47 to 1.94) at the 95^th^ percentile and 1.95 IU/L (95% CI 1.58 to 2.58) at the 97.5^th^ percentile. There was no significant difference in the reference interval between male and female volunteers at either the 95^th^ or 97.5^th^ percentile.

**Table 3:** TRAb reference intervals for healthy Chinese volunteers using the Abbott Alinity system.

	Number of subjects	95^th^ percentile (95% CI)	97.5^th^ percentile (95% CI)
Total	366	1.56 (1.45-1.78)	1.95 (1.58-2.14)
Male	164	1.51 (1.40-2.00)	2.01 (1.47-2.14)
Female	202	1.63 (1.47-1.94)	1.95 (1.58-2.58)

Abbreviations CI: confidence interval.

## Discussion

This study demonstrated that the Abbott Alinity TRAb assay exhibits good analytical performance with verified precision, detection capabilities, and linearity. This assay demonstrated strong correlation and agreement with the well-established Roche Elecsys anti-TSHR assay. ROC analysis confirmed its high sensitivity and specificity in diagnosing GD. The unisex reference interval for the Abbott TRAb assay for Chinese population was also established.

In terms of analytical performance, our results are consistent with previous reports. A study comparing the Abbott TRAb CMIA assay and the Roche TRAb ECLIA assay found that the Abbott assay had good precision, detection capabilities, and linearity, in line with the manufacturer’s claims. Additionally, the Abbott assay showed agreement with the Roche assay in method comparison, with a persistent positive bias of 0.79 IU/L (relative bias: 16.5%) compared to the Roche assay [20]. Another study comparing four different TRAb immunoassays confirmed that the Abbott Alinity assay had acceptable analytical performance with verified linearity, limit of quantification (LoQ), and precision. Concordance analysis showed that the Abbott Alinity assay was in good agreement with the other three TRAb assays (Abbott ARCHITECT, Roche Cobas e411, Thermo Scientific Kruptor), with over 95% overall agreement in all three pair-wise comparisons [27]. As a third-generation competitive immunoassay, the Abbott TRAb assay achieves high analytical sensitivity by utilizing a high-affinity monoclonal antibody (M22) to compete with patient’s endogenous TRAb for binding sites on the TSH receptor [20]. This method, which replaces the labeled TSH used in earlier assay generations, enables the effective detection of TRAb even at low concentrations, resulting in performance comparable to other established third-generation assays [45].

In this study, the Spearman rank correlation coefficient of Snibe vs. Roche (0.784) was significantly lower than that of Abbott vs. Roche (0.972). Bland-Altman analysis demonstrated a negative bias of −10.0% for Snibe vs. Roche, indicating that Snibe’s TRAb assay may produce unreliable low results, especially for sample with medium and high TRAb concentrations. This discrepancy may be attributable methodological differences that both the Abbott and Roche assays employ the human monoclonal stimulating antibody M22 [20,25,46], whereas the Snibe assay utilizes a sandwich immunoassay incorporating TSH receptor-coated nanomagnetic microbeads and a spa-antigen labeled with ABEI [26]. Multiple studies have shown that persistent high TRAb levels after ATD treatment are associated with an increased risk of GD recurrence [15,16,47], and high pre-medication TRAb levels may predict poor response to ATD treatment [16]. Therefore, using a TRAb assay with less bias and can accurately reflect the real situation is important.

In terms of clinical performance, the Abbott TRAb CMIA assay in this study demonstrated high sensitivity (98.4%), specificity (99.4%) and AUC (0.999) for diagnosing GD. Although no prior studies have reported the sensitivity, specificity, or AUC of this specific assay, its performance is comparable to the third-generation Roche anti-TSHR assay, which utilizes the human monoclonal stimulating antibody M22. Previous studies have shown the Roche assay achieved sensitivity of 95–100%, specificity of 95.3 to 99%, and an AUC ranging from 0.98 to 0.995 in diagnosing GD [10,25,46]. The Abbott TRAb assay’s high diagnostic accuracy stems from its CMIA platform. The assay’s biotin-free design eliminates analytical interference from exogenous biotin, preventing falsely low results, while its 6-point calibration ensures high precision across the measuring range [20].

This study established the reference interval for the Chinese population using Abbott Alinity i TRAb assay. The reference interval established was closely aligned with the TRAb threshold reported in a previous epidemiological study in China [6], where TRAb levels above 1.75 IU/L were considered as elevated. Notably, that threshold falls between the 95^th^ percentile (1.56 IU/L) and the 97.5^th^ percentile (1.95 IU/L) upper reference limits determined in this study. Few studies have reported TRAb reference intervals among healthy subjects. Notably, a study from Thailand involving 1,947 healthy individuals found the 97.5^th^ percentile of TRAb (Cobas Roche) to be 1.24 IU/L, with no significant gender differences [35]. These results align with our findings. However, two earlier studies indicated that male patients with GD had higher TRAb levels than female patients [48]. Additionally, TRAb positivity (>2.5 IU/L) was significantly higher among male patients with chronic thyroiditis and hyperthyroidism compared to female patients (P = 0.034) [49]. These discrepancies may stem from the interference of autoantibodies present in serum samples from patients with thyroid diseases and the use of different TRAb assays across studies [48–50]. Such inconsistencies underscore the necessity to determine optimal TRAb cutoffs for different thyroid diseases and to assess whether gender or other factors significantly influence these cutoffs.

## Conclusions

In summary, the Abbott Alinity TRAb CMIA assay exhibited reliable analytical performance, which is consistent with the manufacturer’s claims regarding precision, detection capabilities, and linearity. It demonstrated superior consistency and correlation with the Roche TRAb assay compared to the Snibe TRAb assay, highlighting its advantages in managing GD. It was efficient in diagnosing GD, with an optimal cutoff value of 2.89 IU/L. The reference interval without gender difference for the Abbott Alinity TRAb assay has been established.

This study has certain limitations. This study was designed as a single-center and single-ethnicity study, which may not reflect the impact of regional and ethnic variations. Additionally, the reference interval established in this study can only apply to serum TRAb measurements obtained using the Abbott Alinity i system. Future research should focus on addressing the study’s limitations by conducting large-scale, multicenter investigations involving diverse ethnic and regional populations to validate and potentially refine the established reference interval. Additionally, studies are needed to determine optimal TRAb cutoff values across a broader spectrum of thyroid disorders and to explore the influence of demographic and clinical variables on assay performance.

## Supporting information

S1 Table Comparison table of total test results (N) and critical value observation ratio (P). (DOCX)

S2 Table Inconsistent test results between Abbott and Roche TRAb assay. (DOCX)

S3 Table Inconsistent test results between Snibe and Roche TRAb assay. (DOCX)

S1 Fig Method comparison results for TRAb (IU/L) in the Graves’ disease group, limited to samples within the measuring range. **(A)** Passing-Bablok regression and **(B)** Bland-Altman analysis of Abbott vs Roche; **(C)** Passing-Bablok regression and **(D)** Bland-Altman analysis of Abbott vs Snibe; **(E)** Passing-Bablok regression and **(F)** Bland-Altman analysis of Roche vs Snibe.
(PDF)

S2 Fig Method comparison results for TRAb (IU/L) in the non-hyperthyroidism group, limited to samples within the measuring range. **(A)** Passing-Bablok regression and **(B)** Bland-Altman analysis of Abbott vs Roche; **(C)** Passing-Bablok regression and **(D)** Bland-Altman analysis of Abbott vs Snibe; **(E)** Passing-Bablok regression and **(F)** Bland-Altman analysis of Roche vs Snibe.
(PDF)

S1 Data Raw data underlying the findings of this study. (ZIP)
