# Supplementary material for: Performance evaluation and reference interval establishment of Abbott Alinity thyroid-stimulating hormone receptor antibody (TRAb) assay for diagnosing Graves’ disease
Source: PLoS One. 2026 Feb 4;21(2):e0339494. doi: 10.1371/journal.pone.0339494 (PMC12871968; doi:10.1371/journal.pone.0339494)
Supplement: S1 Table — (DOCX) [file pone.0339494.s001.docx]

**Supplementary Tables**

**S1 Table Comparison table of total test results (N) and critical value observation ratio (P)**

| **Total test results (N)** | **Critical value observation ratio (P)** |
| --- | --- |
| 20 | 85% |
| 30 | 87% |
| 40 | 88% |
| 50 | 88% |
| 60 | 90% |
| 70 | 90% |
| 80 | 90% |
| 90 | 91% |
| 100 | 91% |
| 150 | 92% |
| 200 | 92% |

Excerpted from Health Industry Standard of the People's Republic of China WS/T 514-2017: Establishment and verification of detection capability for clinical laboratory measurement procedures. Available from: <http://www.nhc.gov.cn/ewebeditor/uploadfile/2017/02/20170209183211191.pdf>.
