# Supplementary material for: Performance evaluation and reference interval establishment of Abbott Alinity thyroid-stimulating hormone receptor antibody (TRAb) assay for diagnosing Graves’ disease
Source: PLoS One. 2026 Feb 4;21(2):e0339494. doi: 10.1371/journal.pone.0339494 (PMC12871968; doi:10.1371/journal.pone.0339494)
Supplement: S2 Table — (DOCX) [file pone.0339494.s002.docx]

**Supplementary Tables**

**S2 Table Inconsistent test results between Abbott and Roche TRAb assay**

| **No. of samples** | **Abbott (IU/L)**  **(Cut-off: 3.10 IU/L)** | **Roche (IU/L)**  **(Cut-off: 1.75 IU/L)** | **Snibe (IU/L)**  **(Cut-off: 1.50 IU/L)** |
| --- | --- | --- | --- |
| **Abbott negative and Roche positive** | | | |
| 1 | 2.98 (-) | 2.48 (+) | 1.815 (+) |
| 2 | 3.09 (-) | 2 (+) | 2.003 (+) |
| 3 | 2.54 (-) | 1.94 (+) | 3.005 (+) |
| 4 | 2.66 (-) | 1.9 (+) | 2.476 (+) |
| **Abbott positive and Roche negative** | | | |
| 5 | 3.69 (+) | <0.8 (-) | 1.857 (+) |
| 6 | 4.08 (+) | <0.8 (-) | 0.891 (-) |
| 7 | 3.19 (+) | 1.27 (-) | 1.817 (+) |
