# Supplementary material for: Performance evaluation and reference interval establishment of Abbott Alinity thyroid-stimulating hormone receptor antibody (TRAb) assay for diagnosing Graves’ disease
Source: PLoS One. 2026 Feb 4;21(2):e0339494. doi: 10.1371/journal.pone.0339494 (PMC12871968; doi:10.1371/journal.pone.0339494)
Supplement: S3 Table — (DOCX) [file pone.0339494.s003.docx]

**Supplementary Tables**

**S3 Table Inconsistent test results between Snibe and Roche TRAb assay**

| **No. of samples** | **Abbott (IU/L)**  **(Cut-off: 3.10 IU/L)** | **Roche (IU/L)**  **(Cut-off: 1.75 IU/L)** | **Snibe (IU/L)**  **(Cut-off: 1.50 IU/L)** |
| --- | --- | --- | --- |
| 1 | 1.87 (-) | 1.27 (-) | 1.795 (+) |
| 2 | 1.22 (-) | 0.8 (-) | 1.618 (+) |
| 3 | 1.33 (-) | 0.8 (-) | 1.523 (+) |
| 4 | 1.97 (-) | 0.8 (-) | 1.576 (+) |
| 5 | 3.69 (+) | <0.8 (-) | 1.857 (+) |
| 6 | 0.93 (-) | <0.8 (-) | 1.757 (+) |
| 7 | 1.75 (-) | 1.19 (-) | 1.771 (+) |
| 8 | 3.19 (+) | 1.27 (-) | 1.817 (+) |
| 9 | 2.8 (-) | 1.5 (-) | 1.895 (+) |
| 10 | 2.39 (-) | 1.71 (-) | 1.684 (+) |
