# Supplementary material for: Performance evaluation and reference interval establishment of Abbott Alinity thyroid-stimulating hormone receptor antibody (TRAb) assay for diagnosing Graves’ disease
Source: PLoS One. 2026 Feb 4;21(2):e0339494. doi: 10.1371/journal.pone.0339494 (PMC12871968; doi:10.1371/journal.pone.0339494)
Supplement: S1 Fig — (A) Passing-Bablok regression and (B) Bland-Altman analysis of Abbott vs Roche; (C) Passing-Bablok regression and (D) Bland-Altman analysis of Abbott vs Snibe; (E) Passing-Bablok regression and (F) Bland-Altman analysis of Roche vs Snibe. (PDF) [file pone.0339494.s004.pdf]

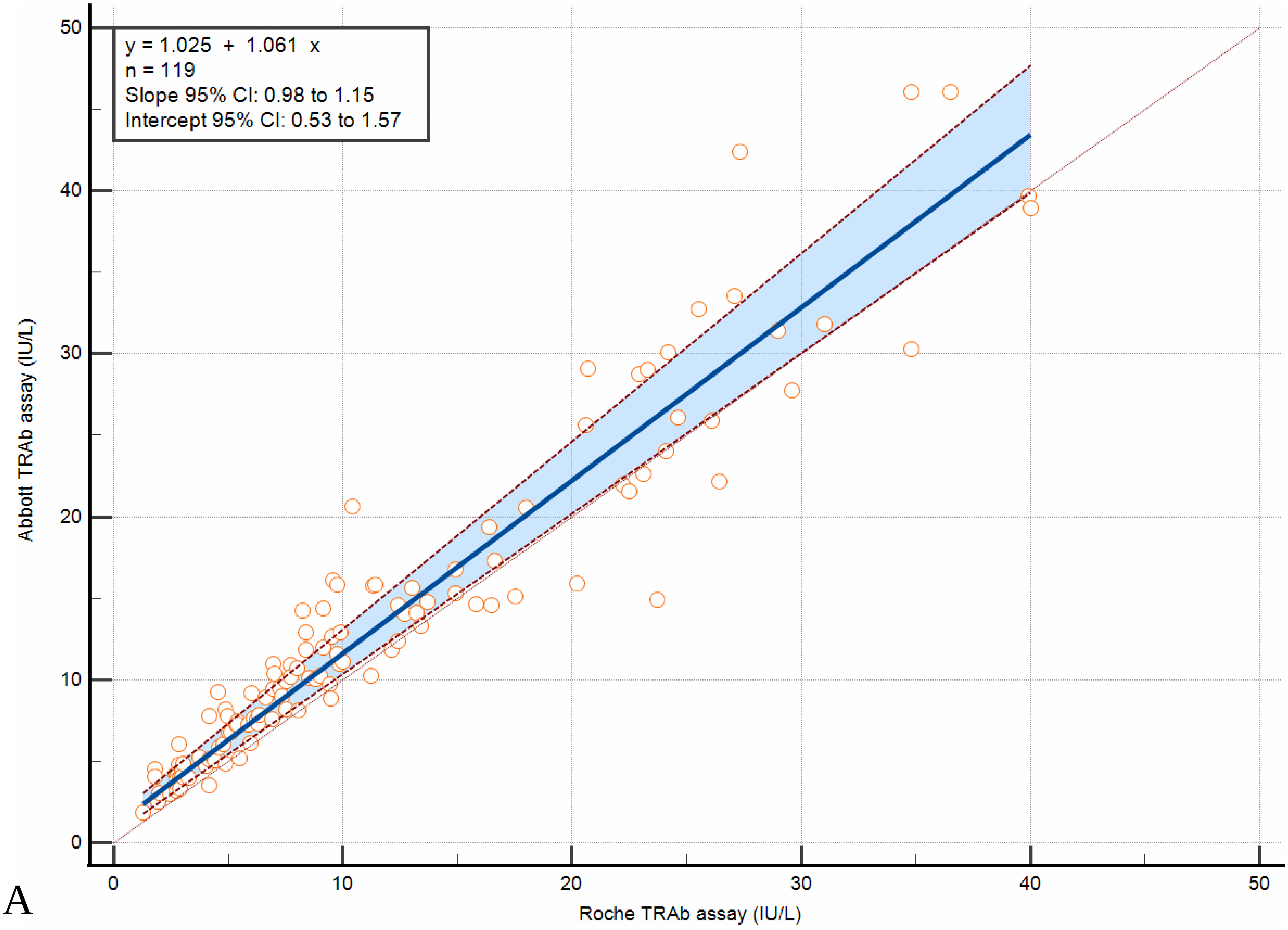

y = 1.025 + 1.061 x
n = 119
Slope 95% CI: 0.98 to 1.15
Intercept 95% CI: 0.53 to 1.57
Abbott TRAb assay (IU/L)
Roche TRAb assay (IU/L)
0
10
20
30
40
50
0
10
20
30
40
50

A

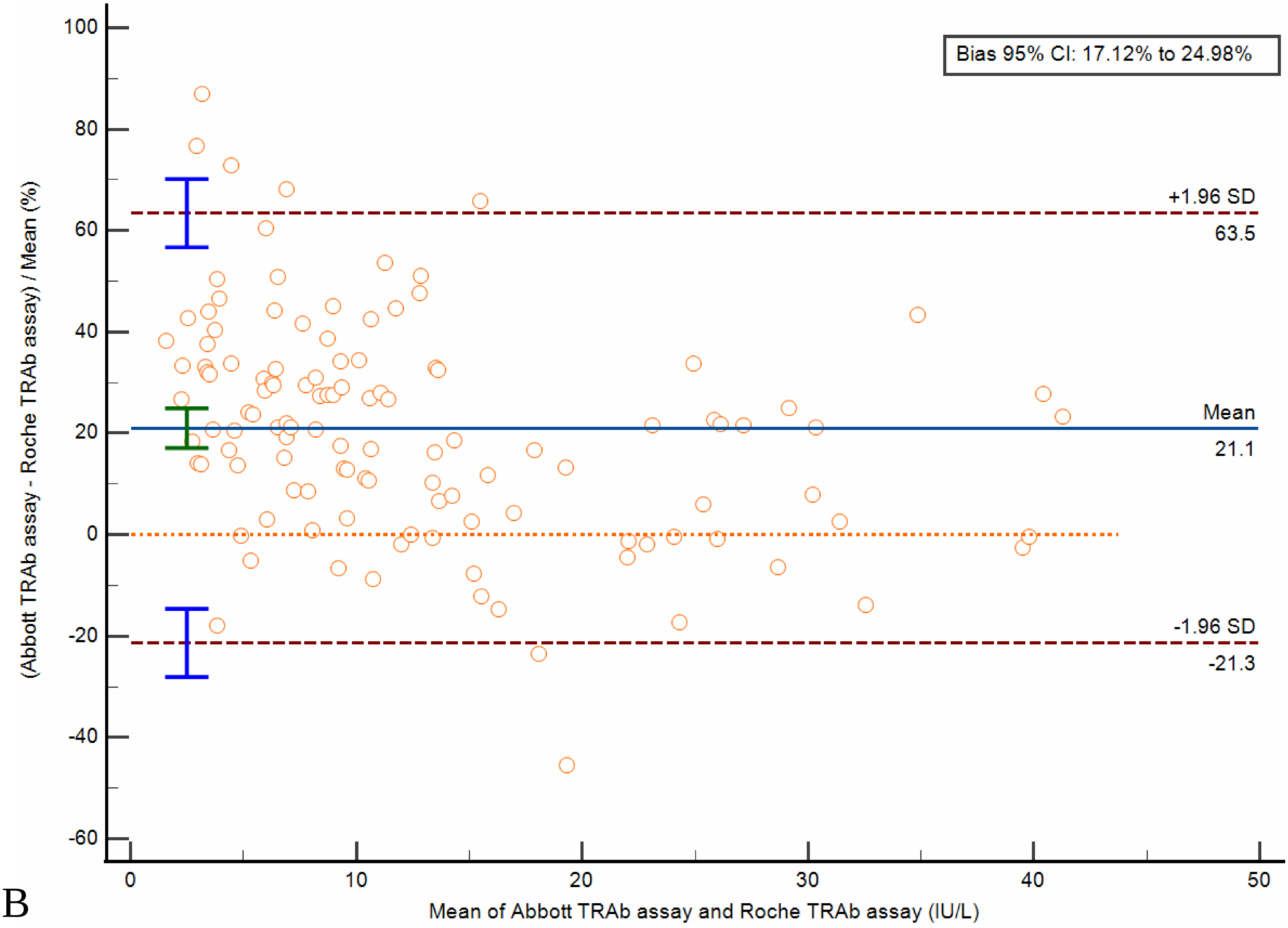

Bias 95% CI: 17.12% to 24.98%
+1.96 SD
63.5
Mean
21.1
-1.96 SD
-21.3
(Abbott TRAb assay - Roche TRAb assay) / Mean (%)
Mean of Abbott TRAb assay and Roche TRAb assay (IU/L)
100
80
60
40
20
0
-20
-40
-60
0
10
20
30
40
50

B

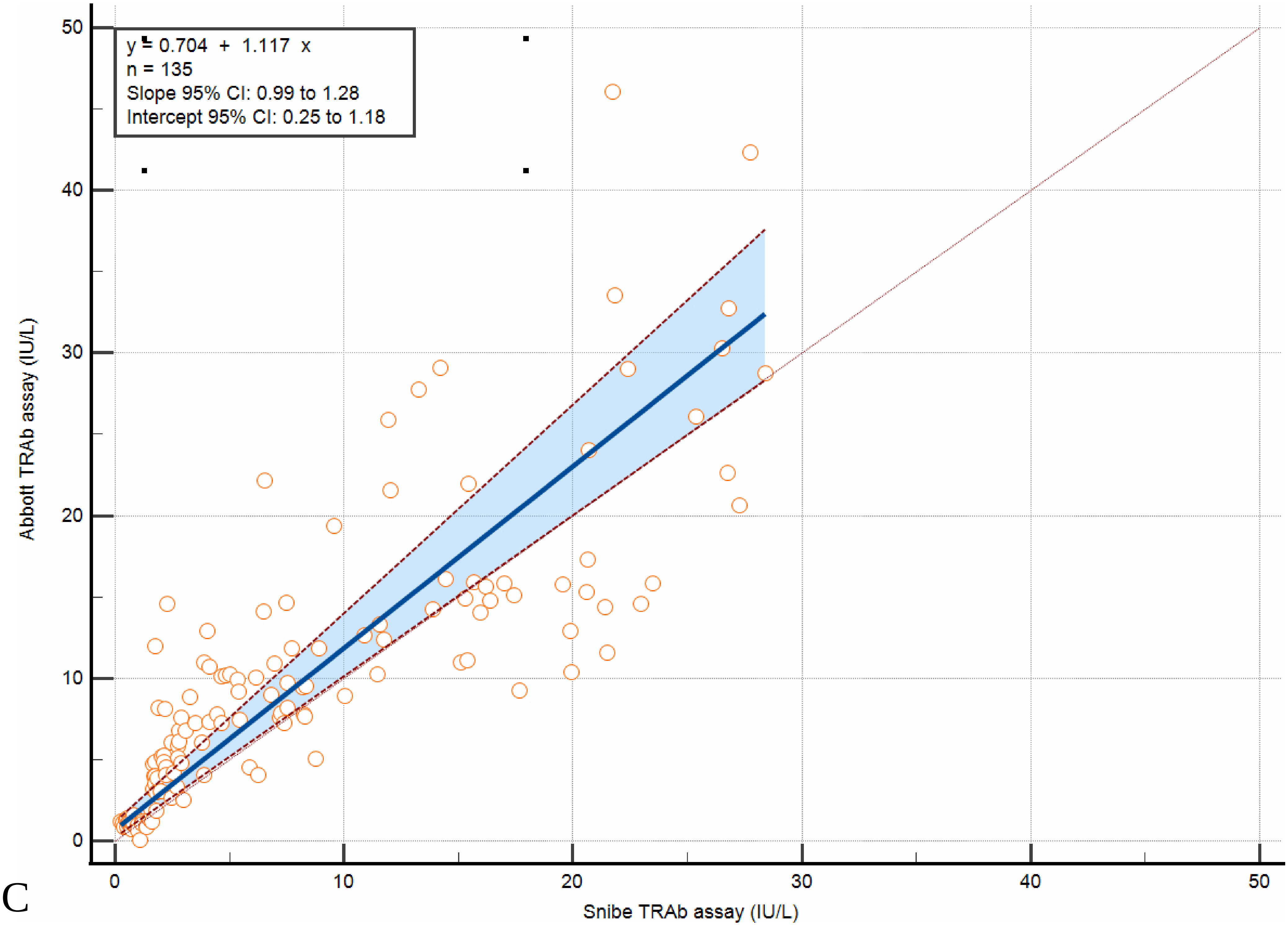

y = 0.704 + 1.117 x
n = 135
Slope 95% CI: 0.99 to 1.28
Intercept 95% CI: 0.25 to 1.18
Abbott TRAb assay (IU/L)
Snibe TRAb assay (IU/L)
0
10
20
30
40
50

C

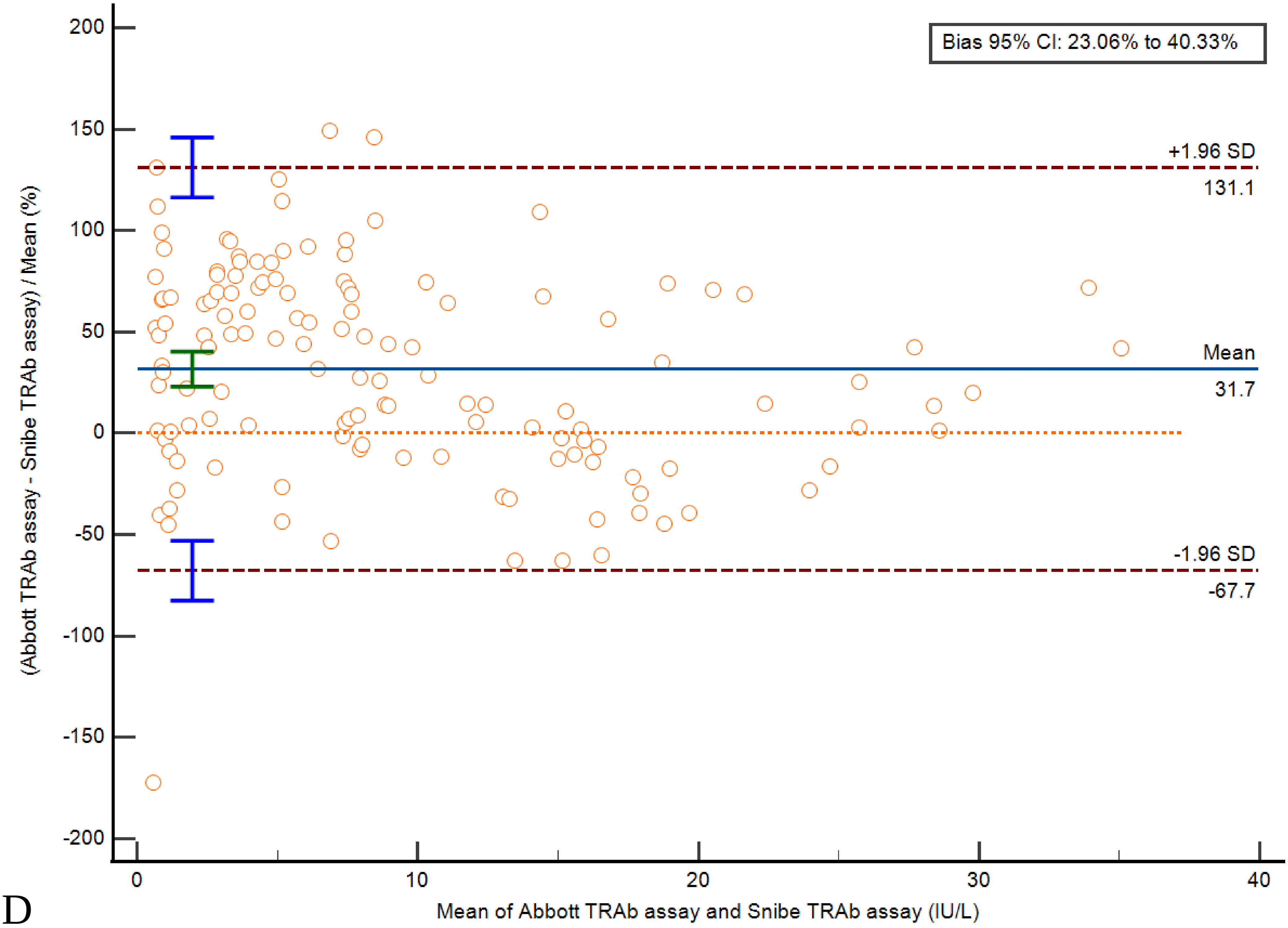

Bias 95% CI: 23.06% to 40.33%
+1.96 SD
131.1
Mean
31.7
-1.96 SD
-67.7
200
150
100
50
0
-50
-100
-150
-200
0
10
20
30
40
(Abbott TRAb assay - Snibe TRAb assay) / Mean (%)
Mean of Abbott TRAb assay and Snibe TRAb assay (IU/L)

D

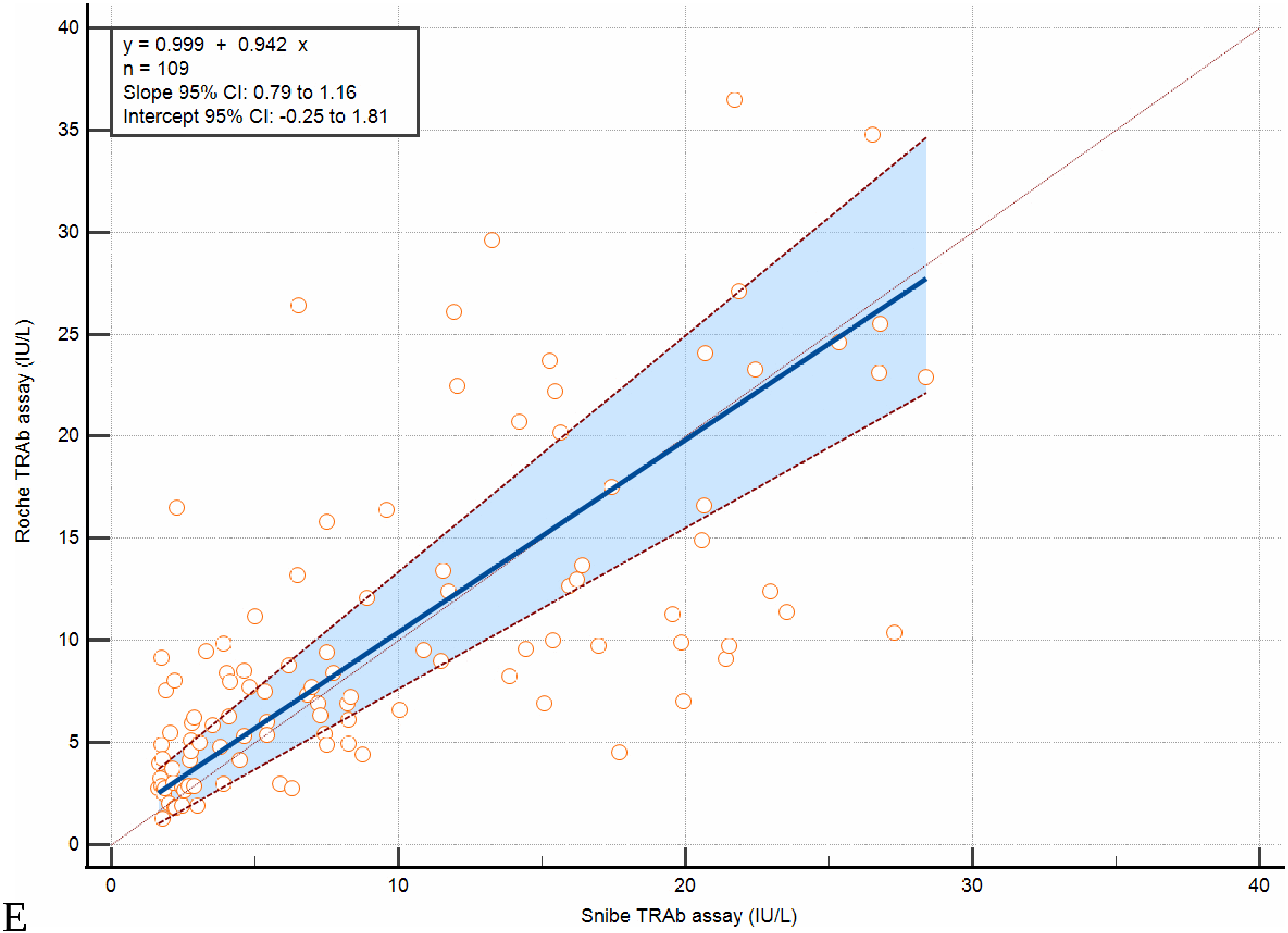
y = 0.999 + 0.942 x
n = 109
Slope 95% CI: 0.79 to 1.16
Intercept 95% CI: -0.25 to 1.81
Roche TRAb assay (IU/L)
Snibe TRAb assay (IU/L)
0
5
10
15
20
25
30
35
40
0
10
20
30
40

E

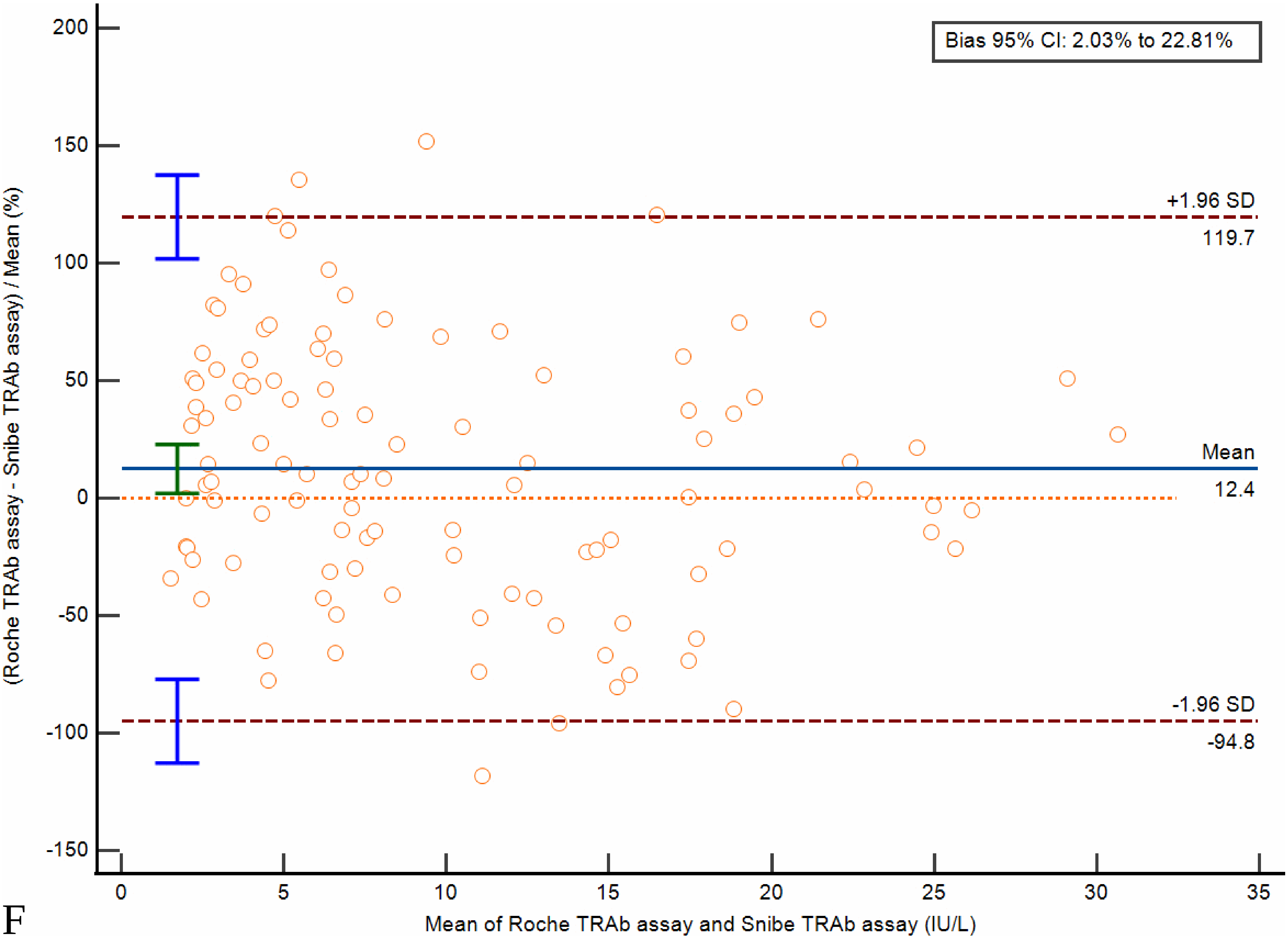

Bias 95% CI: 2.03% to 22.81%
+1.96 SD
119.7
Mean
12.4
-1.96 SD
-94.8
(Roche TRAb assay - Snibe TRAb assay) / Mean (%)
200
150
100
50
0
-50
-100
-150
0
5
10
15
20
25
30
35
Mean of Roche TRAb assay and Snibe TRAb assay (IU/L)

F
