# Supplementary figures and images for: Performance evaluation and reference interval establishment of Abbott Alinity thyroid-stimulating hormone receptor antibody (TRAb) assay for diagnosing Graves’ disease
Source: PLoS One. 2026 Feb 4;21(2):e0339494. doi: 10.1371/journal.pone.0339494 (PMC12871968; doi:10.1371/journal.pone.0339494)

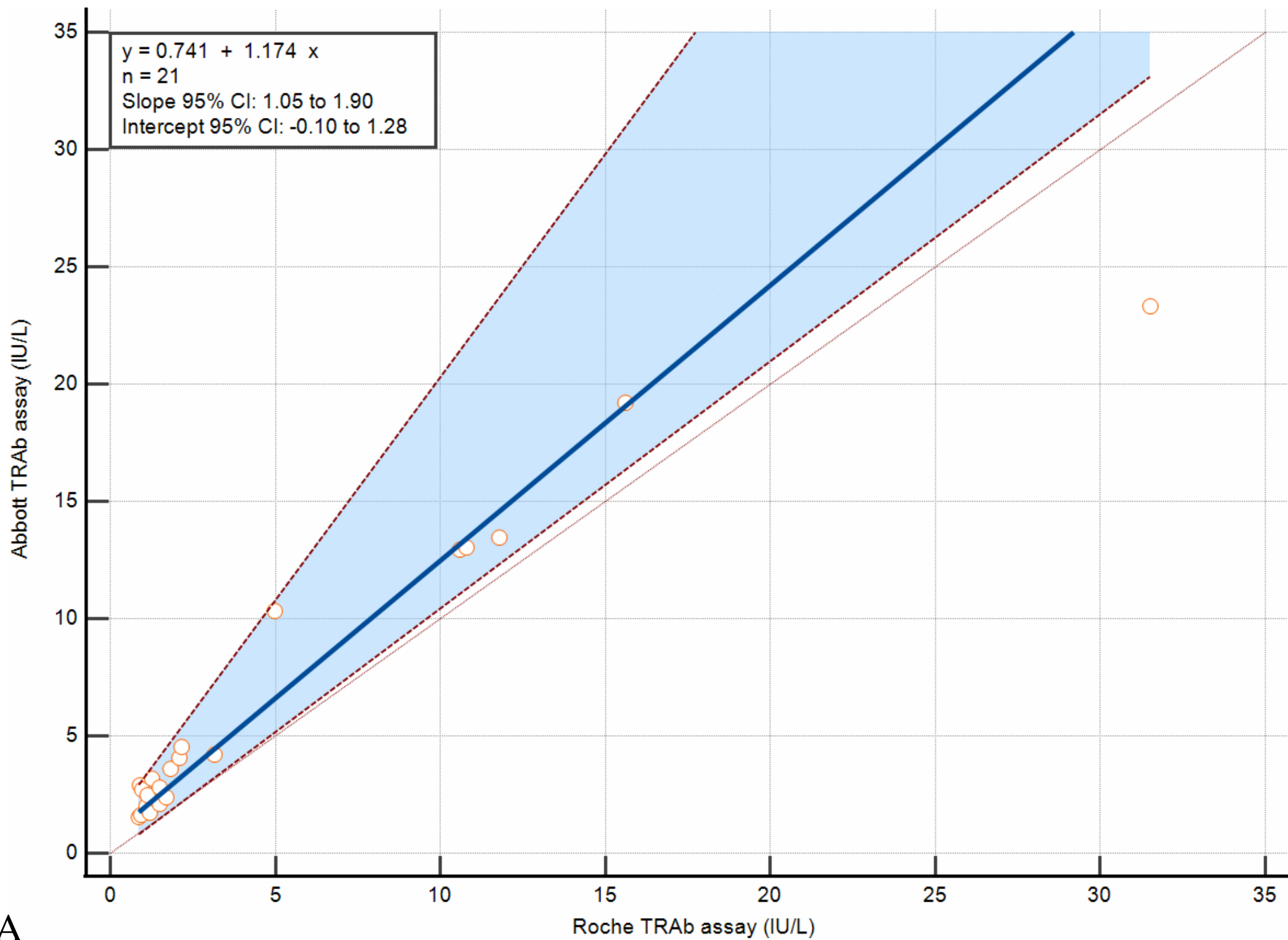

A

B

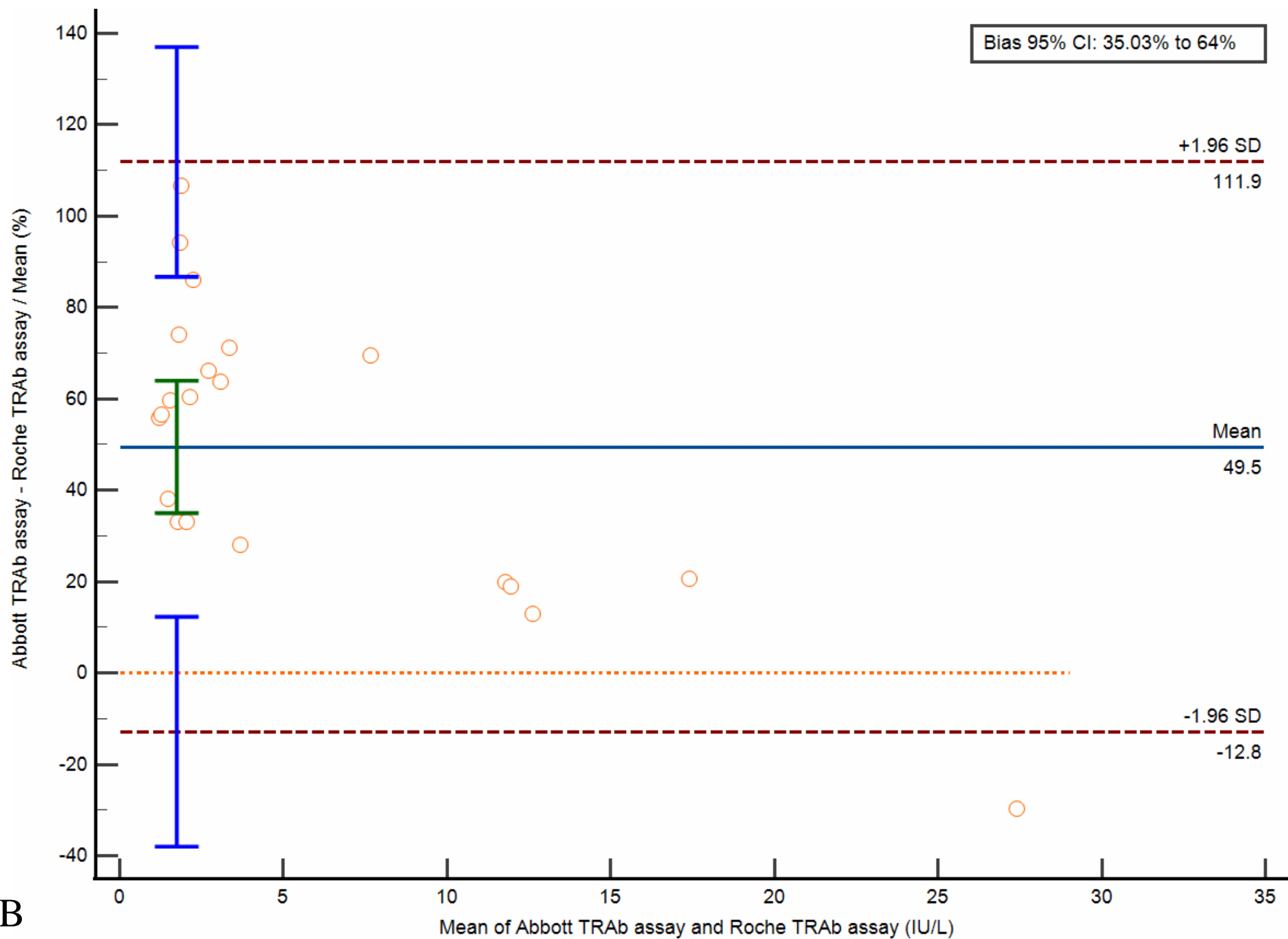

C

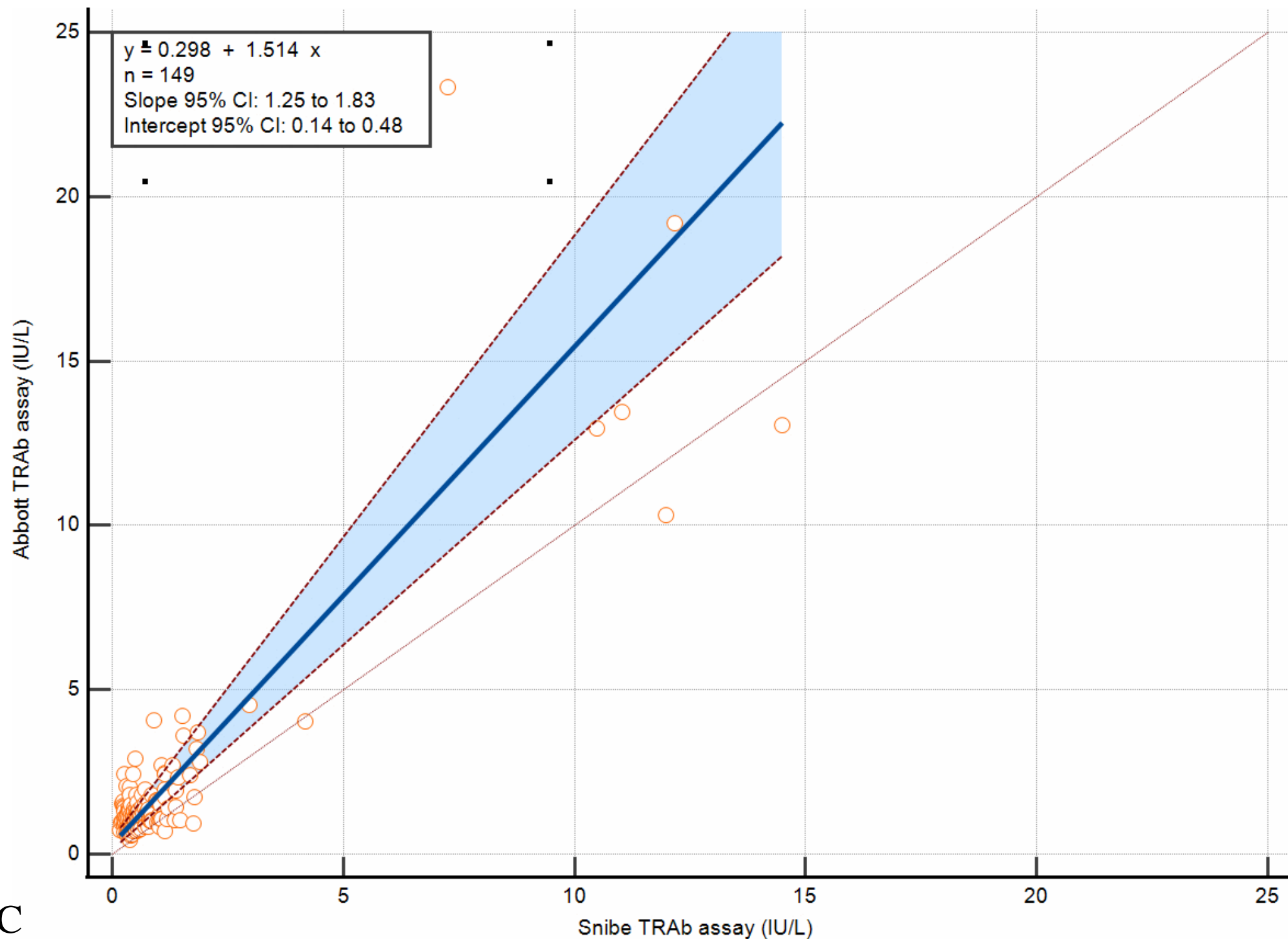

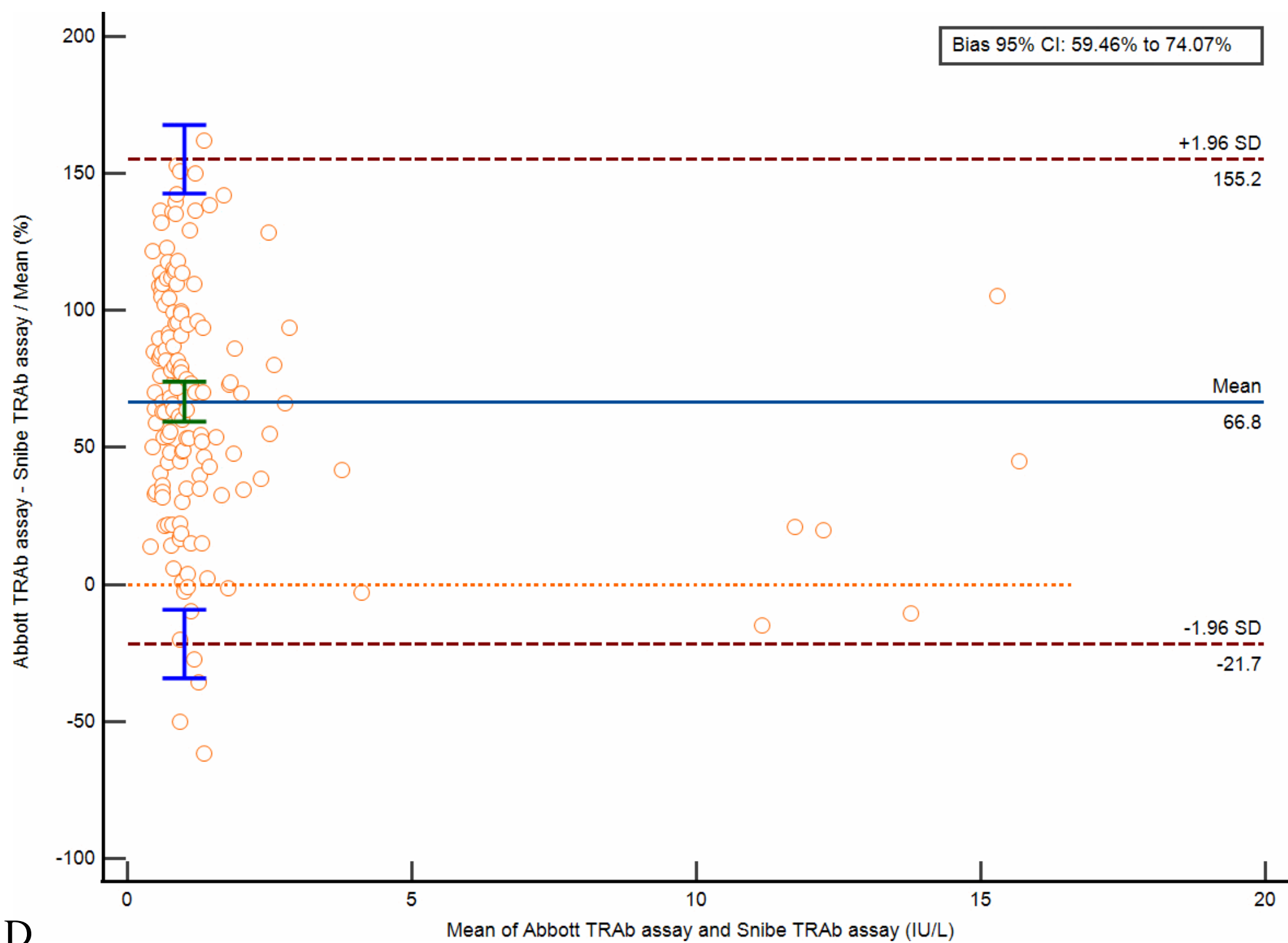

E

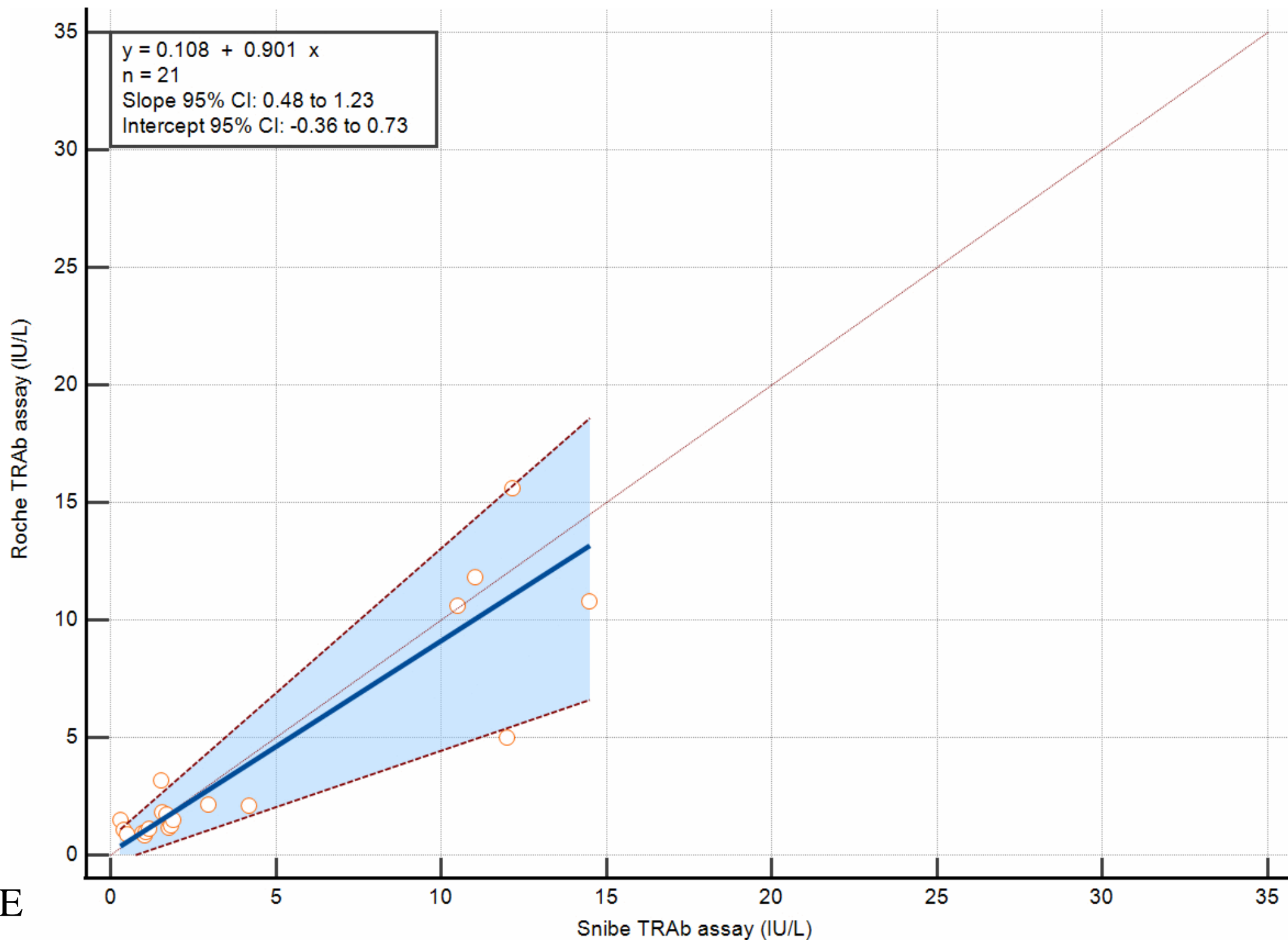

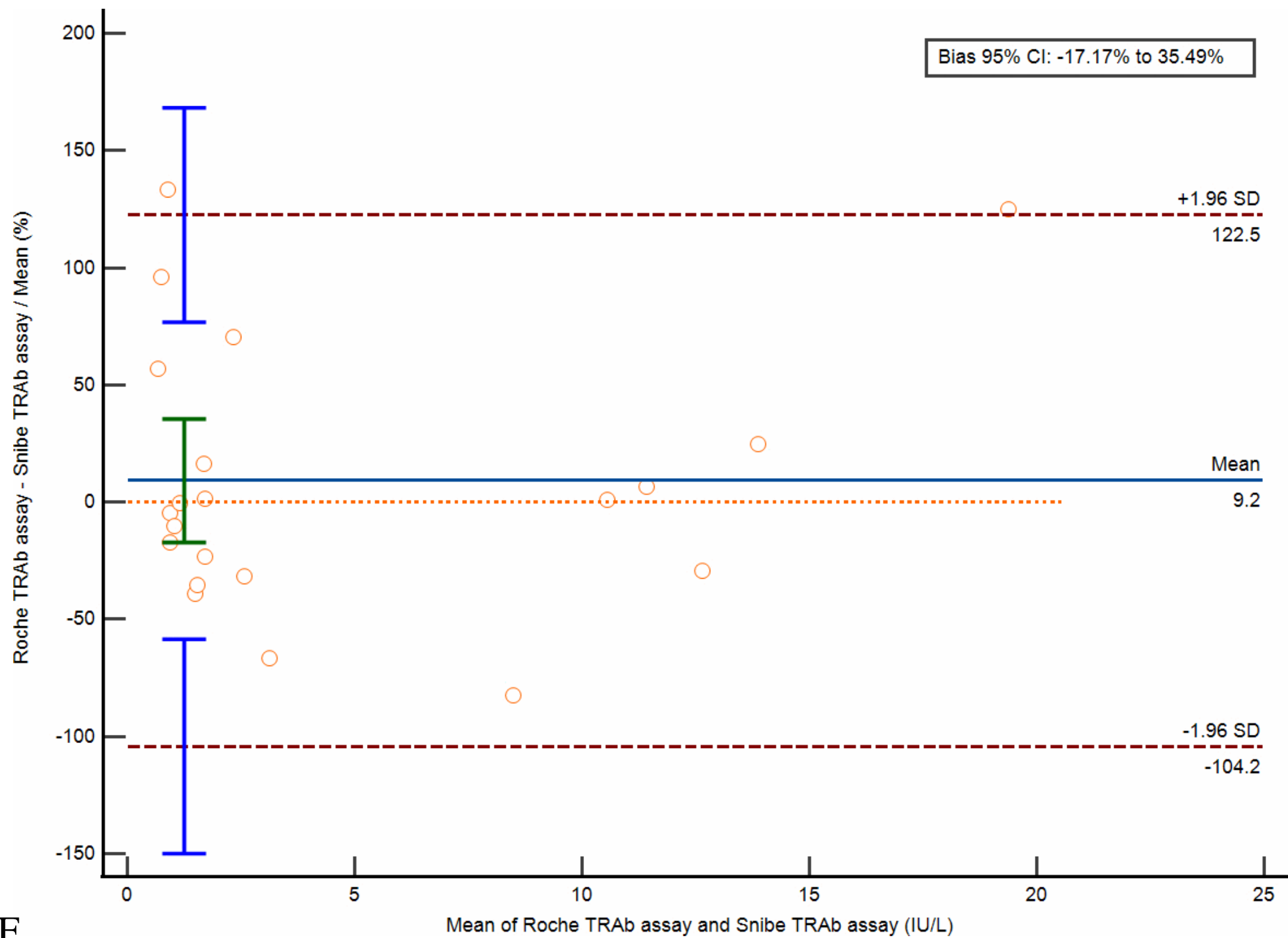

Supplement: S2 Fig — (A) Passing-Bablok regression and (B) Bland-Altman analysis of Abbott vs Roche; (C) Passing-Bablok regression and (D) Bland-Altman analysis of Abbott vs Snibe; (E) Passing-Bablok regression and (F) Bland-Altman analysis of Roche vs Snibe. (PDF) [file pone.0339494.s005.pdf]
